# Evaluation of a mobile behavior change program for weight loss in breast cancer survivors

**DOI:** 10.1038/s41523-024-00659-x

**Published:** 2024-06-29

**Authors:** Sherry Shen, Erica Salehi, Charlie White, Yuan Chen, Neil M. Iyengar

**Affiliations:** 1 Memorial Sloan Kettering Cancer Center, New York, NY USA; 2 Weill Cornell Medicine, New York, NY USA

**Keywords:** Breast cancer, Risk factors

## Abstract

Post-diagnosis weight gain is common in early-stage breast cancer and is associated with increased risk of recurrence and mortality. Intentional weight loss is difficult to maintain, and digital lifestyle interventions may provide a scalable approach to address this challenge. In this prospective single-arm study (ClinicalTrials.gov NCT04753268; February 15, 2021), key eligibility criteria included: stage I–III breast cancer, body mass index (BMI) ≥ 27.5 kg/m^2^, and completion of cancer treatment ≥6 months before study enrollment. Participants were provided with a behavioral change mobile application (Noom®). The primary endpoint was a change in self-reported weight from baseline to 26 weeks. Secondary endpoints included engagement, changes in physical activity, dietary patterns, and patient-reported outcomes (PRO). In total, 31 patients were enrolled (mean age 56.8 ± 9.9, mean baseline BMI 33.5 kg/m^2^ ± 6.5). The mean weight change was −4.8 kg ( ± 4.4, *P* < 0.001), mean percent weight change was −5.6% ( ± 5.0%); 11/31 patients (35.5%) lost ≥5% of their initial weight. Metrics of digital application engagement associated with weight loss ≥5% included articles read (*P* = 0.012), weights logged (*P* = 0.006), food records logged (*P* = 0.001), messages sent (*P* = 0.001), and application open count (*P* = 0.014). Significant increases were seen in mean daily step count (*P* = 0.004), GPAQ scores (*P* = 0.002), and Body Image Scale scores (*P* < 0.001). Mean energy intake remained consistently in a calorie-restricted range of 1300–1400 kcal/day. In this study, breast cancer survivors were highly engaged with a behavioral change smartphone application which led to clinically significant weight loss, increased physical activity, maintenance of an energy-restricted diet, and improvements in body image.

## Introduction

With improvements in treatment in the last few decades, the majority of women with early-stage breast cancer ultimately transition to survivorship; there are nearly 4 million breast cancer survivors in the United States as of January 2022, a number that is expected to increase over time^1,2^. Obesity is a leading modifiable risk factor for breast cancer recurrence and mortality among breast cancer survivors^3–5^. Following diagnosis, 50–96% of women experience weight gain, which is more common among younger patients and those treated with chemotherapy^6–8^. Post-diagnosis weight gain ≥5% of baseline body weight or increases in body mass index (BMI) ≥2.0 kg/m^2^ are associated with increased risks of recurrence (RR 1.53, 95% CI 1.04–2.24), breast cancer death (RR 1.64, 95% CI 1.04–2.24), and all-cause mortality (HR 1.59, 95% CI 1.12–2.27)^9,10^.

Several randomized controlled trials have tested the feasibility and short-term efficacy of lifestyle interventions for weight loss and/or maintenance of optimal weight in breast cancer survivors. The majority of these trials have tested behavioral modification via telephone-based counseling to encourage a healthy dietary pattern and physical activity. Participants randomized to the intervention arms experienced weight loss in the range of 3.7–5.6 kg or 5–6% of baseline weight over a range of 6–12 months^11–16^. However, notable variability in weight loss efficacy and durability has been reported. For example, in the Women’s Intervention Nutrition Study (WINS), women who received telephone-based counseling for dietary fat reduction had significantly more weight loss (difference of 2.7 kg, *P* = 0.005) at 12 months and improved relapse-free survival (HR 0.76, 95% CI 0.60–0.98) after a median follow-up of 60 months compared to the control group, although the degree of weight reduction achieved diminished at 3 and 6 years^11,17^. Conversely, in the Women’s Healthy Eating and Living (WHEL) trial testing a telephone-based counseling program, there were no significant differences in weight loss or event-free survival HR 0.91, 95% CI 0.71–1.15) between the intervention and control arms^12^. There are several potential reasons for the inconsistent weight and survival outcomes across these telephone-based lifestyle intervention trials, including differences in cohort baseline characteristics, time from diagnosis to enrollment, and breast cancer characteristics. Nonetheless, these findings raise the possibility that the impact of lifestyle interventions on breast cancer outcomes may be mediated by weight loss.

Intentional weight loss following breast cancer diagnosis is challenging to achieve and maintain. Interactive virtual platforms such as smartphone mobile applications have been shown to induce weight loss in the range of −0.1 to −3.0 kg and BMI reduction in the range of −0.1 to −1.0 kg/m^2^ ^18^. However, the majority of commercially available applications do not use evidence-based strategies for weight loss, such as the inclusion of self-monitoring capabilities, goal-setting, motivational strategies, or personalized feedback^19^. The Noom program is a digital, multi-behavioral intervention that utilizes cognitive behavioral therapy, motivational interviewing, self-determination theory, and behavior change techniques to promote lifestyle changes delivered via a smartphone mobile application^20–22^. Use of the Noom application has been reported to improve diet quality leading to weight loss and reductions in total body fat, visceral fat, and waist circumference, and improvements in metabolic factors such as blood pressure, fasting glucose, and lipids^23–27^. Observational data suggest that the program also promotes weight loss, physical activity, healthy eating habits, and quality of life among individuals that reported a history of cancer^28–30^. Additional preliminary data supporting the use of smartphone apps for weight loss and maintenance in cancer populations are promising but sparse. In a retrospective analysis of 107 cancer survivors, 70 of whom had breast cancer, use of the Noom app resulted in mean 4.7 kg (6.2%) weight loss at 4 months^30^. In a randomized controlled pilot trial of 65 patients with colorectal polyps, use of the Noom app led to a greater reduction in weight at 3 months compared to control (−1.25 vs. −0.42 kg, *P* < 0.01)^28^. Similarly, the Noom intervention improved nutritional status and quality of life at 3 months compared to usual care among patients with pancreatic cancer in a randomized trial^29^. On the basis of these findings, we investigated the effects of the Noom intervention on weight, physical activity, and patient-reported outcomes (PROs) among overweight and obese breast cancer survivors in a prospective single-arm trial.

## Results

**Fig. 1:**
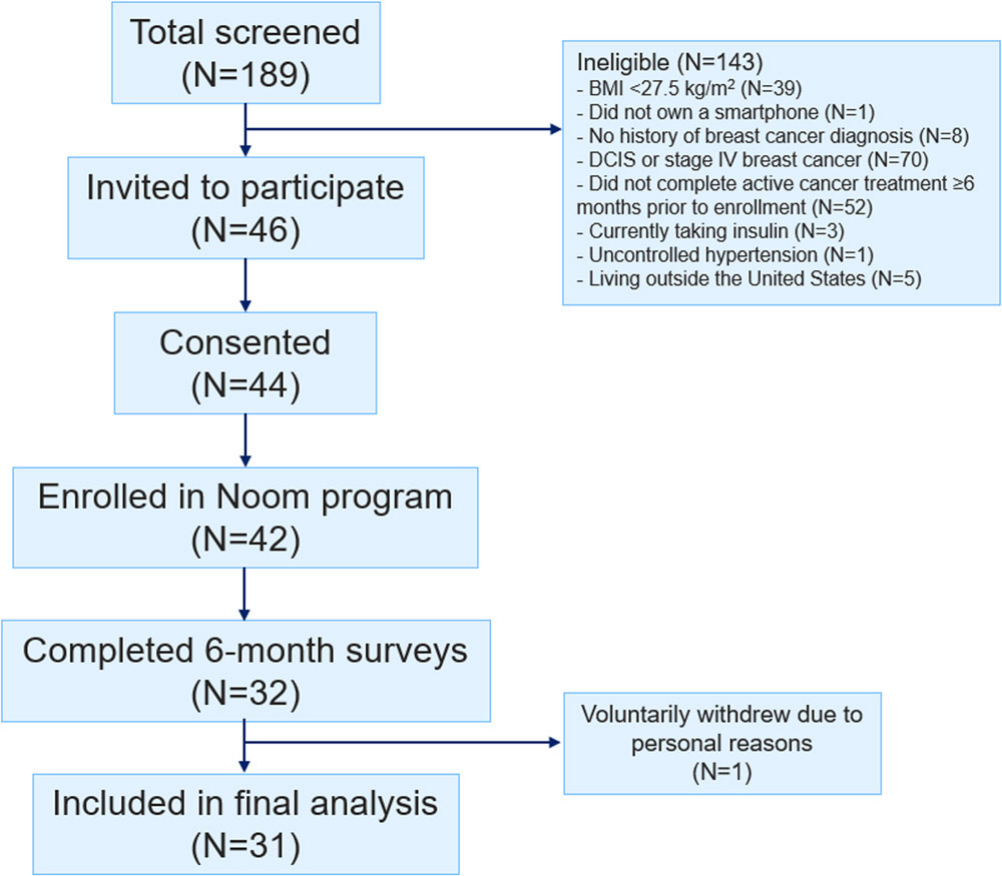
CONSORT diagram. Flowchart of participant disposition in this study.

A total of 31 patients were included in this study; 24 were recruited via the Dempsey Center, and 7 were recruited via social media (Fig. 1). Baseline characteristics of the cohort are shown in Table 1. The mean age was 56.8 (SD 9.9) years; mean baseline weight was 88.4 kg (SD 13.8) and mean baseline BMI was 33.5 kg/m^2^ (SD 6.5). At enrollment, the mean time since breast cancer diagnosis was 4.7 years (SD 5.9). 20 patients (64.5%) received chemotherapy, 25 (80.6%) had or were currently receiving endocrine therapy, and 3 (9.7%) had received anti-HER2 therapy. Patient baseline characteristics were balanced between recruitment methods except for stage at diagnosis (*P* = 0.04).

**Table 1:** Baseline characteristics of the study cohort

Recruitment method				
Characteristic	Overall, *N* = 31^a^	Dempsey Center, *N* = 24^a^	Social media, *N* = 7^a^	*P*^b^
Age				0.8
Mean (SD)	56.8 (9.9)	56.6 (9.9)	57.7 (10.6)	
Race				0.4
White	29 (94%)	23 (96%)	6 (86%)	
Black	2 (6.5%)	1 (4.2%)	1 (14%)	
Marital status				0.4
Never married	2 (6.7%)	1 (4.3%)	1 (14%)	
Married/living with partner	22 (73%)	18 (78%)	4 (57%)	
Divorced/separated	6 (20%)	4 (17%)	2 (29%)	
Unknown	1	1	0	
Education				0.7
High school diploma/GED	4 (13%)	4 (17%)	0 (0%)	
Some college or associate degree	7 (23%)	5 (21%)	2 (29%)	
College graduate/graduate degree	20 (65%)	15 (63%)	5 (71%)	
Initial weight				>0.9
Mean (SD)	88.4 (13.8)	88.7 (15.0)	87.6 (9.6)	
BMI				0.5
Mean (SD)	33.5 (6.5)	33.9 (6.4)	31.9 (7.1)	
Years since breast cancer diagnosis				0.1
Mean (SD)	4.7 (5.9)	3.3 (3.5)	9.3 (9.5)	
Unknown	1	1	0	
Stage at diagnosis				**0.04**
1	10 (34%)	7 (32%)	3 (43%)	
2	14 (48%)	13 (59%)	1 (14%)	
3	5 (17%)	2 (9.1%)	3 (43%)	
Unknown	2	2	0	
Lumpectomy	14 (45%)	12 (50%)	2 (29%)	0.4
Unilateral mastectomy	4 (13%)	3 (13%)	1 (14%)	>0.9
Bilateral mastectomy	13 (42%)	9 (38%)	4 (57%)	0.4
Radiation	22 (71%)	18 (75%)	4 (57%)	0.4
Chemotherapy	20 (65%)	14 (58%)	6 (86%)	0.4
Prior or current endocrine therapy	25 (81%)	20 (83%)	5 (71%)	0.6
Prior anti-HER2 therapy	3 (9.7%)	3 (13%)	0 (0%)	>0.9

^a^*n* (%).

^b^Wilcoxon rank-sum test; Fisher’s exact test.

Significant *P* values are shown in bold.

### Weight change

**Fig. 2:**
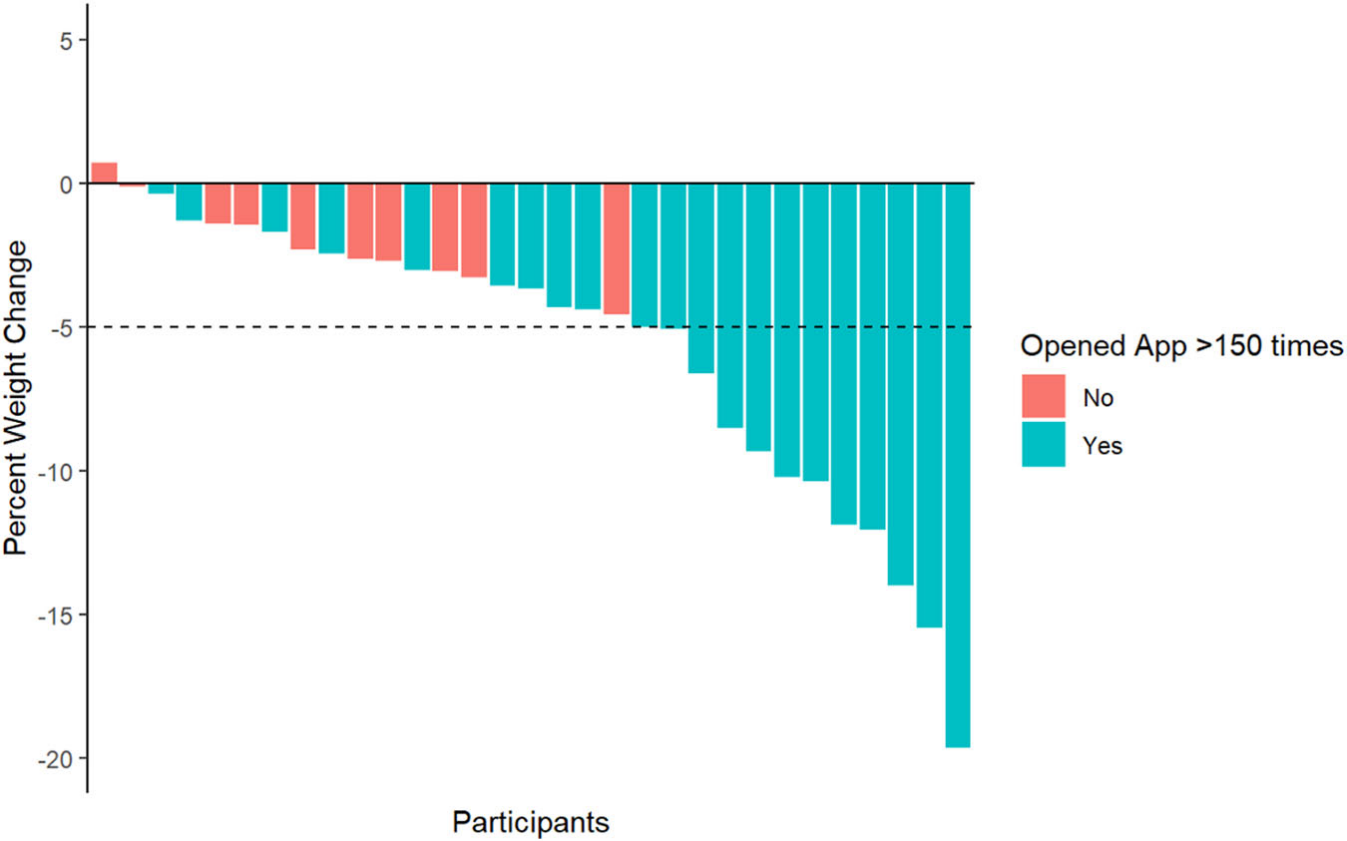
Waterfall plot of percent weight change for individual patients. Weight change is stratified by mean number of times the mobile application was opened during the study period (mean 150, SD 40).

**Fig. 3:**
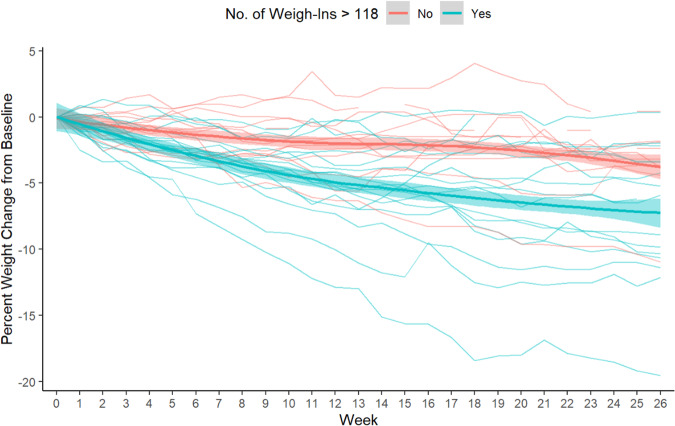
Spaghetti plot of percent weight change for individual patients. Weight change was stratified by mean number of weights logged (mean 118, SD 56).

The mean weight at the conclusion of the 26-week study period was 83.6 kg (SD 14.6). The mean weight change was −4.8 kg (SD 4.4, *P* < 0.001). The mean percent weight change was −5.6% (SD 5.0%); 11 out of 31 patients (35.5%) lost ≥5% of enrollment weight. Engagement metrics with the Noom mobile platform are summarized in Table 2. Study participants opened the Noom application a mean of 150 times (SD 40), read a mean of 429 articles (SD 254) out of a total of 775 articles, logged a mean of 118 weights (SD 57), logged a mean of 528 meals (SD 284), logged a mean of 48 exercises (SD 62), and sent a mean of 64 messages to their coach (SD 44). Among engagement metrics, greater number of total articles read, total weights logged, total meals logged, messages sent to coaches, and times the Noom mobile application was opened were all significantly associated with weight loss ≥5% (*P* < 0.05; Table 2). Per participant percent weight change stratified by the cohort means for the number of times the mobile application was opened, and the number of weigh-ins are shown in Figs. 2 and 3, respectively.

**Table 2:** Weight loss and engagement with the Noom program over the 26-week study period

% Weight loss ≥ 5%				
Characteristic	Overall, *N* = 31^a^	No, *N* = 20^a^	Yes, *N* = 11^a^	*P*^b^
Total articles read	429 (254)	337 (252)	598 (158)	**0.01**
Total number of weights logged	118 (57)	98 (52)	155 (47)	**0.01**
Number of meals logged	528 (284)	407 (254)	749 (194)	**0.001**
Percent calories from green food items	21 (8)	18 (7)	25 (9)	0.1
Percent calories from yellow food items	38 (7)	39 (8)	37 (5)	0.3
Percent calories from red food items	41 (10)	43 (10)	39 (8)	0.4
Number of exercises logged	48 (62)	46 (60)	52 (68)	0.4
Number of messages sent to coach	64 (44)	45 (30)	99 (44)	**0.001**
Number of times mobile application opened	150 (40)	136 (44)	175 (9)	**0.01**
Sum score for group activities	149 (203)	103 (170)	232 (238)	0.1

^a^Mean (SD).

^b^Wilcoxon rank-sum test; Wilcoxon rank-sum exact test.

Significant *p* values are shown in bold.

### Dietary intake

Food records at T1 and T2 were available from 25/31 (80.5%) patients. Dietary energy restriction was consistent starting during week 1 through the end of the intervention period (Table 3) and was within the calorie-restricted range of 1200–1500 kcal/day recommended for weight loss in women with overweight or obese BMI^31^. There were no significant changes in total carbohydrate intake, or total protein intake (Table 3) during the intervention. Proportional fat intake increased by 4.0% (95% CI 0.7–7.3%, *P* = 0.03), including a 1.5% increase in proportional monounsaturated fatty acid intake (95% CI 0.4–2.7%, *P* = 0.03). There were no significant changes in HEI total score and food group intake (Supplementary Table 1).

**Table 3:** Dietary changes from T1 to T2

Characteristic	T1^a^	T2^a^	*P*^b^
Total grams	1408.1 (399.6)	1231.6 (392.3)	0.1
Energy (kcal)	1302.9 (185.8)	1337.6 (322.2)	>0.9
Fat (g)	52.6 (12.2)	59.3 (14.5)	**0.045**
Carbohydrates (g)	146.6 (37.2)	146.4 (50.2)	>0.9
Protein (g)	66.5 (20.7)	61.4 (16.5)	0.2
% Calories from fat	35.1 (6.3)	39.1 (5.8)	**0.024**
% Calories from carbohydrates	43.4 (9.2)	41.4 (6.8)	0.2
% Calories from protein	20.6 (5.6)	18.7 (4.1)	0.1
% Calories from alcohol	0.9 (3.0)	0.8 (2.3)	0.9
% Calories from SFA	11.0 (3.2)	12.4 (2.8)	**0.048**
% Calories from MUFA	11.6 (2.5)	13.1 (2.7)	**0.026**
% Calories from PUFA	9.1 (3.7)	10.0 (3.7)	0.3
Total daily whole grain ounce equivalents	1.1 (0.9)	1.2 (1.2)	0.5
Added sugars (by total sugars)	24.3 (23.0)	21.2 (17.2)	0.8
Total dietary fiber (g)	17.8 (3.8)	17.0 (5.1)	0.5
Saturated fat	16.5 (6.0)	18.7 (5.3)	0.1
Cholesterol	273.2 (183.4)	204.6 (136.4)	**0.045**
Total daily servings of fruit	1.9 (1.7)	1.2 (1.1)	0.1
Total daily servings of vegetables	3.3 (1.4)	3.2 (1.8)	0.9
Red and processed meats	1.4 (1.9)	1.2 (1.2)	0.4

^a^Mean (SD).

^b^Wilcoxon signed-rank exact test; Wilcoxon signed-rank test with continuity correction.

Significant *p* values are shown in bold.

### Physical activity

All patients completed the Global Physical Activity Questionnaire (GPAQ) at baseline and 26 weeks. The mean baseline GPAQ score for physical activity was 990.3 (SD 1371.4); only 14/31 patients (45.2%) met the World Health Organization physical activity recommendations at the time of enrollment^32^. By 26 weeks, the mean score improved to 1773 (SD 1409, *P* = 0.002) and 23/31 patients (74.2%, 95%CI 56.8–86.3%) met WHO guidelines (29.0 percentage point increase, *P* = 0.03). 20/31 (64.5%) patients had complete step count data at week 1 and week 26. At baseline, mean daily step count for week 1 of study participation was 2744.4 (SD 2194.2) and increased to 4245 (SD 2703.0) at week 26 (*P* = 0.001) (Table 4).

**Table 4:** Change in physical activity measures from baseline to 26 weeks

Characteristic	Baseline^a^	26 weeks^a^	*P*^b^
GPAQ score	990 (1371)	1773 (1409)	0.002
Daily step count	2744 (2194)	4245 (2703)	0.001

^a^Mean (SD).

^b^Wilcoxon signed-rank test.

### Patient-reported outcomes

Several PRO scores improved from baseline to 26 weeks (Table 5). Improvements were found in Body Image Scale (mean change −3.9, SD 5.4, *P* < 0.01), and Weight Management Support Appraisal subscale (mean change +2.3, SD 3.1, *P* < 0.01).

**Table 5:** Change in patient-reported outcome measures from baseline to 26 weeks

Characteristic	Baseline^a^	26 weeks^a^	Difference	95% CI	*P*^b^	SD of mean difference^c^
PROMIS-Fatigue T score (Complete Pairs)^d^	47.8 (6.0)	46.1 (6.8)	−1.7	−4.5, 1.1	0.2	6.7
Unknown	7	7				
PROMIS-Depression T Score	48.1 (7.2)	47.1 (7.1)	−1.0	−3.1, 1.2	0.4	5.9
PROMIS-Anxiety T Score	49.3 (9.3)	48.2 (8.9)	−1.1	−3.8, 1.6	0.5	7.5
PROMIS-Physical Functioning T Score	46.8 (6.4)	48.9 (7.9)	2.1	0.1, 4.2	0.1	5.7
Body Image Scale	11.7 (7.4)	7.8 (7.0)	−3.9	−5.9, −2.0	**<0.001**	5.4
WMSI Emotional	11.8 (3.4)	11.0 (3.5)	−0.8	−1.9, 0.2	0.1	2.8
WMSI Instrumental	15.6 (4.8)	17.0 (5.2)	1.4	0.1, 2.6	0.1	3.4
WMSI Informational	12.5 (4.5)	12.4 (4.1)	−0.2	−1.5, 1.1	0.7	3.5
WMSI Appraisal	10.8 (4.0)	13.1 (5.2)	2.3	1.1, 3.4	**0.001**	3.1

*CI* confidence interval.

^a^Mean (SD).

^b^Wilcoxon signed-rank test.

^c^SD.

^d^Among 24 participants with a score at both time points.

Significant *p* values are shown in bold.

## Discussion

In this single-arm study in survivors of primary breast cancer, use of a behavioral change smartphone application led to an average weight loss of nearly 6% over a 26-week intervention period. The mobile intervention also led to maintenance of caloric restriction and improvements in physical activity levels and patient-reported body image and weight management support. Higher levels of engagement with the mobile intervention, such as more frequent use of the application, was associated with ≥5% weight loss.

The degree of weight loss in this prospective trial is consistent with or greater than results from behavioral intervention trials that tested telephone-based counseling in breast cancer survivors. In the WINS trial (*n* = 48,835), women with a history of early-stage breast cancer randomized to the low-fat diet counseling arm had greater mean weight loss compared with the usual care arm (difference 2.2 kg, SD 8.4); notably, 27.3% of the study population had BMI ≥ 30 kg/m^2^ at baseline^33^. In contrast, no significant difference in weight change was reported in the WHEL trial which randomized 3088 women with early-stage breast cancer to a telephone counseling dietary intervention aimed at increasing fruit, vegetable, and fiber intake and decreasing fat intake compared with usual care; only 12.2% of patients in the WHEL trial had BMI ≥30 kg/m^2^ at baseline^12^. In the Lifestyle Intervention in Adjuvant Treatment of Early Breast Cancer (LISA) study, which randomized 338 women with early-stage breast cancer to telephone-based diet and physical activity counseling or usual care, mean weight change was −5.3% (SD 4.9) in the intervention arm versus −0.7% (SD 4.9) in the usual care arm at 6 months; 57.3% of patients in this study had BMI > 30 kg/m^2^ (see ref. ^14^). Finally, in the Breast Cancer Weight Loss (BWEL) study which randomized 3181 women with early-stage breast cancer to a telephone-based intervention promoting caloric restriction and increased physical activity, mean weight change was −4.8% (SD 7.9) in the intervention arm at 12 months; mean baseline BMI was 34.5 kg/m^2^ (see ref. ^34^). Findings from these and other trials have established the feasibility of telephone-based weight loss interventions in breast cancer survivors.

Few studies have tested smartphone-based weight loss interventions in breast cancer survivors. A study of 33 breast cancer survivors with BMI ≥ 25 kg/m^2^ tested a hospital-specific smartphone tool and demonstrated mean weight change of −0.9 kg (range −4.8 kg to +1.8 kg) over 4 weeks^35^. Another study of ten breast cancer survivors tested Mobile health (mHealth) self-monitoring of diet behaviors via daily text messages, wireless device tracking, and four motivational interviewing-based phone sessions, and resulted in mean weight change of −1.5 kg (SD 3.5) over 10 weeks^36^. A third study of ten breast cancer survivors implemented the MapMyFitness mobile application combined with a social media health education intervention; weight changed from a mean baseline of 79.5 kg (SD 20.8) to 77.2 kg (SD 21.7, mean change −2.3 kg) over 10 weeks^37^. Finally, a feasibility study including 80 breast cancer survivors with BMI ≥25 kg/m^2^ tested the Energy Balance on Cancer (BENECA) mHealth application and resulted in mean weight change of −1.42 kg (95% CI −1.97 to −0.87, *P* < 0.001) over 8 weeks^38^. Our findings demonstrate substantial weight loss using the Noom application that is comparable to historical telephone-or counseling-based interventions and greater weight reduction over a longer study period than other published smartphone application trials in breast cancer survivors.

Although weight gain following breast cancer diagnosis is associated with worse breast cancer outcomes, it remains unclear whether weight loss improves outcomes^9^. Only the WINS trial demonstrated improvement in recurrence-free survival (HR 0.85, 95% CI 0.74–0.96) in the intervention arm whereas the WHEL and LISA studies did not result in significant differences in survival outcomes between the intervention and control arms^11^. Heterogeneity in trial design, intervention, and degree of weight loss likely contributed to mixed results from these trials. The BWEL trial aims to address this knowledge gap. While weight change at 12 months in the BWEL trial has been reported, this study is powered to assess invasive disease-free survival and follow-up is ongoing. Smartphone applications may be more convenient and accessible than telephone-based interventions, with the potential to increase engagement and promote long-term adherence. Future studies testing smartphone applications with large sample sizes powered for breast cancer-specific survival endpoints are needed.

Engagement metrics in our trial compare favorably with prior studies in mixed or other cancer populations^28–30^. In a retrospective cohort that included survivors of any cancer, the number of weekly meals logged (median 26, IQR 19–33), articles read (median 25, IQR 11–28), and messages sent to coaches (median 2, IQR 0–3) were similar to usage metrics in our trial population^30^. Greater usage of the application was associated with achieving ≥5% weight loss in our study, which is consistent with data from patients with colorectal polyps^28^. Tailoring mobile applications to the needs and experiences of cancer survivors may further enhance adherence, interaction, and weight loss efficacy and durability^39^. While comfort with technology may be a concern with smartphone-based interventions, particularly among older patients, among all patients screened for this study, only 1 was excluded due to not owning a smartphone.

In our study, physical activity measures improved significantly both by patient-reported GPAQ and by pedometer step count with use of the Noom application. Total energy intake did not significantly change from T1 to T2 and was consistent with a restricted daily caloric intake goal for weight loss. Dietary patterns were evaluated via food logs within the Noom application; baseline intake prior to enrollment was not assessed. Underreporting and decreased data quality over time are known limitations of food records; in our study, 25/31 participants had complete food log data at both timepoints for analysis^40,41^. In addition, the use of a single dietary assessment instrument limited our ability to draw conclusions about the contribution of diet quality and caloric intake as drivers of weight loss as compared to physical activity. A combination of validated instruments to assess diet such as a food frequency questionnaire may help to clarify diet-related results in future studies with similar designs. Further research should also address methods to improve reporting adherence among patients that do not achieve a target weight loss in order to better tailor the intervention.

Improvement in patient-reported physical function in this trial is consistent with findings from other lifestyle intervention trials in cancer survivors. In a study of 100 breast cancer survivors who were recruited within 6 months of completing their adjuvant cancer treatment, an aerobic and resistance exercise intervention significantly improved quality of life (QOL) as measured by the Functional Assessment of Cancer Therapy-Breast (FACT-B) and SF-36 scales after 16 weeks^42^. Similarly, a study of 174 breast cancer survivors who were enrolled at a mean of 2.8 years after adjuvant therapy showed that patients enrolled in the two exercise dosing regimen arms had significant improvements in FACT-B scores compared with the attention control arm^43^. However, in another study that included 112 breast cancer survivors, the majority of whom were >2 years from diagnosis, baseline QOL was already high, and a 6-month supervised exercise intervention did not significantly affect QOL measures compared to usual care^44^. In our study, the lack of significant improvements in most QOL scores may be due to longer time from breast cancer diagnosis (4.7 years), since symptom burden related to breast cancer and its treatment is anticipated to improve over time. PRO improvements may also differ by degree of weight loss experienced^45^. Nonetheless, participants in our study reported significant improvements in Body Image Scale scores. Thus, our findings suggest that smartphone applications that are tailored to cancer survivors could offer a scalable lifestyle intervention to improve some PROs in addition to weight loss. Additional studies are needed to assess whether the smartphone application improves additional QOL parameters if provided closer to the time of breast cancer diagnosis.

Findings from this study should be interpreted within the context of its limitations. First, the trial was designed as a pilot study to determine effect sizes in a heterogeneous population of breast cancer survivors. Since the study resulted in a significant and clinically meaningful effect size, future randomized trials that are powered to weight loss efficacy or cancer-specific outcomes are warranted. Second, nutrition and some physical activity data were limited by patient self-report or input into the mobile application as discussed above; logging of food intake declined during the study, and complete data were not available for all patients. Strengths of this study include daily weight recording via a linked home scale, multiple assessments of engagement, and convenience of the mobile intervention.

In summary, breast cancer survivors were highly engaged with a behavioral change smartphone application, which led to clinically significant weight loss, maintenance of an energy-restricted diet, increased physical activity, and improvements in body image and perceived support of weight management. Results from this trial could provide oncology care teams with a convenient and scalable intervention for weight loss in breast cancer survivors.

## Methods

### Study design, eligibility, and recruitment

This was a prospective single-arm pilot study that tested a smartphone application for weight loss over an intervention period of 26 weeks in overweight and obese patients with a history of primary breast cancer (ClinicalTrials.gov NCT04753268; February 15, 2021). Eligibility criteria included age ≥18, BMI ≥27.5 kg/m^2^, histologic diagnosis of stage I–III breast cancer, completion of active treatment (surgery, chemotherapy, radiation) ≥6 months prior to study enrollment, and ownership of a smartphone compatible with Noom’s mobile application. Key exclusion criteria included current or recent pregnancy (<6 months postpartum), insulin use, uncontrolled hypertension, or living outside the United States.

Patients were recruited between March 2021 and October 2021 at the Dempsey Center in Maine and via social media. At the Dempsey Center, potentially eligible patients were sent a recruitment letter in which they were directed to a screening survey to assess eligibility if interested. Social media recruitment occurred via posts in breast cancer survivor support groups, and potential patients completed the same screening questionnaire to assess eligibility. This study was approved by an independent Institutional Review Board (Advarra, Inc.), and all participants provided written informed consent. This study complied with all relevant ethical regulations including the Declaration of Helsinki.

### Overall goals of the intervention

The primary goal of the intervention was weight loss at a rate of 0.2–0.9 kg per week. This goal was pursued through dietary energy restriction and the adoption of physical activity guidelines. Daily caloric intake goals, which factored in total energy expenditure, were provided via the smartphone application and were based upon the Harris-Benedict equation^46^. Physical activity goals were consistent with the American Society of Clinical Oncology and American College of Sports Medicine guidelines^47,48^. The intervention is commercially available via a smartphone app (Noom, Inc., New York, NY) and was not altered for this study.

### Intervention content and delivery

The intervention curriculum is grounded in cognitive behavioral therapy, motivational interviewing, and self-determination theory to promote multi-behavior change and improve well-being parameters including diet, exercise, sleep hygiene, and stress management^21,22,49,50^. Approximately 3–5 short articles requiring 1–2 min to read were assigned to participants on a daily basis. Virtual health coaching in the app was provided by registered dietitians; this included individualized support via in-app messages at least once per week. A social network feature allowed participants to post personal challenges, solicit feedback from other users of the app, and respond to others’ challenges.

Participants were encouraged to log all foods and beverages consumed daily and track daily caloric intake via a food color grading system based on calorie density which categorizes foods as low (green), moderate (yellow), or high (red) caloric density^51^. Consuming low caloric density meals leads to reductions in overall energy consumption in obese and non-obese populations independent of portion size^52–58^. Further, diets characterized by foods low in caloric density have been associated with reduced adiposity and lower glycemic load^59–61^. Participants were encouraged to derive 30%, 45%, and 25% of their daily calories from the low, moderate, and high caloric density groups, respectively.

Physical activity was tracked daily via manual exercise logging features or via pedometer from either the mobile device or a personally owned wearable device with Bluetooth device integration. Participants were encouraged to implement predefined, dynamic physical activity goals that built incrementally on starting activity levels to reach ≥150 min per week of moderate to vigorous-intensity exercise or ≥75 min per week of vigorous-intensity exercise and resistance training 2 days per week.

### Outcomes and assessments

The primary endpoint of this study was change in self-reported weight from baseline to the end of the intervention period (26 weeks). Secondary endpoints included: in-app engagement/activity, and changes in physical activity, dietary patterns, and patient-reported outcomes (PROs). At baseline, participants completed a self-report questionnaire that assessed body weight, health behaviors, and demographic information including race, ethnicity, and socioeconomic status. Participants were provided a home scale that was linked to the mobile application to log daily body weight values. Self-reported weight and health behaviors were reassessed via questionnaire at the end of the 26-week intervention period.

Engagement with the mobile application was measured by total number of articles read, number of weights logged, number of meals logged, physical activity logged, number of messages sent to RD coaches, and a sum score for group engagement that was calculated based on interactions with other Noom users.

Dietary intake was assessed at two timepoints: during the first 4 weeks after study registration (T1) and during the final 13–26 weeks of the study (T2). Food logs from 2 non-consecutive days were included in each assessment if the following completeness criteria were met: (1) at least three meals logged and (2) total energy intake range of 500–3500 kcal/day^62,63^. Average intake of nutrients, food group servings, and Healthy Eating Index (HEI) 2015 scores were calculated for both timepoints using the Nutrition Data System for Research (NDSR) software version 2022 (University of Minnesota, MN)^64^. Mean HEI scores at each timepoint were calculated according to the National Cancer Institute scoring algorithm^65^.

Physical activity levels were assessed at baseline and completion of the intervention via the GPAQ and pedometer step count. The GPAQ measures intensity, duration, and frequency of three domains of physical activity (occupational, transport-related, and discretionary/leisure time) and has been validated for assessing change in physical activity levels^66^. Total pedometer step count was recorded in the app during week 1 and week 26 and reported as daily average for those weeks.

Several validated PRO instruments were administered at study entry and conclusion. Assessments included: Patient-Reported Outcomes Measurement Information System (PROMIS) short form questionnaires (PROMIS-Fatigue 8a, PROMIS Emotional Distress-Depression 8a, PROMIS Emotional Distress-Anxiety 8a, and PROMIS-Physical Functioning 8b); the Body Image Scale; and the Weight Management Support Inventory including emotional, instrumental, information, and appraisal subscales^67–69^.

### Statistical analyses

This study was designed as a pilot study to determine effect sizes and characterize patient experience with the mobile intervention. Patient characteristics were summarized overall and compared by recruitment method. Categorical and continuous variables were compared using Fisher’s exact test and Wilcoxon rank-sum test, respectively. Wilcoxon signed-rank test was used to assess changes in weight, physical activity measures, nutrition measures, and PROs. Associations between various baseline factors and engagement factors with weight loss of ≥5% was also evaluated by the Wilcoxon rank-sum test. A two-sided 0.05 significance level was used for all statistical tests. Statistical analyses were performed using R (v. 4.2.2).

### Supplementary information

Supplementary Table 1. Change in Healthy Eating Index (HEI) scores from T1 to T2.

## Data Availability

The datasets generated and/or analyzed during this study are available from the corresponding author upon reasonable request.
