# Supplementary material for: Evaluation of a mobile behavior change program for weight loss in breast cancer survivors
Source: NPJ Breast Cancer. 2024 Jun 29;10:53. doi: 10.1038/s41523-024-00659-x (PMC11217495; doi:10.1038/s41523-024-00659-x)
Supplement: Supplementary file 1 — Supplementary Table 1. Change in Healthy Eating Index (HEI) scores from T1 to T2. [file 41523_2024_659_MOESM1_ESM.pdf]

Supplementary Table 1. Change in Healthy Eating Index (HEI) scores from T1 to T2.

Characteristic	Maximum score	T1[1]	T2[1]	*P*[2]
HEI total score	100	64 (9)	61 (15)	0.56
Total vegetables	5.0	4.5 (0.9)	4.1 (1.2)	0.26
Total green and beans	5.0	3.1 (2.3)	3.4 (2.1)	0.59
Total fruit	5.0	3.2 (1.8)	2.4 (1.8)	0.13
Whole fruit	5.0	4.0 (1.7)	3.3 (1.7)	0.21
Whole grains	10.0	5.2 (3.7)	5.3 (4.1)	0.64
Dairy	10.0	4.7 (2.9)	5.5 (3.2)	0.24
Total protein foods	5.0	4.7 (0.9)	4.8 (0.6)	>0.99
Seafood and plant protein	5.0	3.3 (2.4)	3.0 (2.2)	0.89
Fatty acid ratio	10.0	5.2 (4.0)	5.4 (3.7)	0.84
Sodium	10.0	3.3 (3.0)	3.4 (3.0)	>0.99
Refined grains	10.0	7.4 (3.1)	6.9 (3.6)	0.56
Added sugar	10.0	8.6 (2.5)	9.2 (1.4)	0.38
Saturated fats	10.0	6.2 (3.3)	4.7 (3.0)	0.10

[1]Mean (SD)
[2]Wilcoxon signed rank exact test
